# Social and racial inequalities in diabetes and cancer in the United States

**DOI:** 10.3389/fpubh.2023.1178979

**Published:** 2023-07-19

**Authors:** Nour Massouh, Ayad A. Jaffa, Hani Tamim, Miran A. Jaffa

**Affiliations:** ^1^Epidemiology and Population Health Department, Faculty of Health Sciences, American University of Beirut, Beirut, Lebanon; ^2^Department of Biochemistry and Molecular Genetics, Faculty of Medicine, American University of Beirut, Beirut, Lebanon; ^3^Division of Endocrinology, Diabetes and Metabolic Diseases, Department of Medicine, Medical University of South Carolina, Charleston, SC, United States; ^4^Biostatistics Unit, Faculty of Medicine, Clinical Research Institute, American University of Beirut, Beirut, Lebanon; ^5^College of Medicine, Alfaisal University, Riyadh, Saudi Arabia

**Keywords:** behavioral risk factor surveillance system, cancer, diabetes, health equity, social determinants of health

## Abstract

**Background:**

Cancer and diabetes are among the leading causes of morbidity and mortality worldwide. Several studies have reported diabetes as a risk factor for developing cancer, a relationship that may be explained by associated factors shared with both diseases such as age, sex, body weight, smoking, and alcohol consumption. Social factors referred to as social determinants of health (SDOH) were shown to be associated with the risk of developing cancer and diabetes. Despite that diabetes and social factors were identified as significant determinants of cancer, no studies examined their combined effect on the risk of developing cancer. In this study, we aim at filling this gap in the literature by triangulating the association between diabetes, indices of SDOH, and the risk of developing cancer.

**Methods:**

We have conducted a quantitative study using data from the Behavioral Risk Factor Surveillance System (BRFSS), whereby information was collected nationally from residents in the United States (US) with respect to their health-related risk behaviors, chronic health conditions, and the use of preventive services. Data analysis using weighted regressions was conducted on 389,158 study participants.

**Results:**

Our findings indicated that diabetes is a risk factor that increases the likelihood of cancer by 13% (OR 1.13; 95%CI: 1.05–1.21). People of White race had higher odds for cancer compared to African Americans (OR 0.44; 95%CI: 0.39–0.49), Asians (OR 0.27; 95%CI: 0.20–0.38), and other races (OR 0.56; 95%CI: 0.46–0.69). The indices of SDOH that were positively associated with having cancer encompassed unemployment (OR 1.78; 95%CI: 1.59–1.99), retirement (OR 1.54; 95%CI: 1.43–1.67), higher income levels with ORs ranging between 1.16–1.38, college education (OR 1.10; 95%CI: 1.02–1.18), college graduates (OR 1.31; 95%CI: 1.21–1.40), and healthcare coverage (OR 1.44; 95%CI: 1.22–1.71). On the other hand, the indices of SDOH that were protective against having cancer were comprised of renting a home (OR 0.86; 95%CI: 0.79–0.93) and never married (OR 0.73; 95%CI: 0.65–0.81).

**Conclusion:**

This study offers a novel social dimension for the association between diabetes and cancer that could guide setting strategies for addressing social inequities in disease prevention and access to healthcare.

## 1. Introduction

Cancer and diabetes are among the leading causes of morbidity and mortality from non-communicable diseases (NCDs) in the world (1). In the United States (US), cancer and diabetes accounted for more than 600,000 and 100,000 deaths, respectively, in 2020 (2). Considering these high morbidity and mortality rates, both diseases are posing a heavy burden on the healthcare system. The lifetime risk of developing both cancer and diabetes is 15% (3), with 8–18% of cancer cases being reported as having diabetes (4). An analysis of the National Health Interview Surveys showed that 15.7% of adults with diabetes have had cancer, whereas 13.4% of adults without diabetes have had cancer (5). Along the same lines, an increase in the incidence of cancer was detected in a large cohort of diabetic subjects (Standardized Incidence Ratio 1.16; 95% CI: 1.15–1.16) (6), and in meta-analyses of observational studies which highlighted the association of diabetes with increased total cancer incidence (Random Effects 1.10; 95% CI: 1.04–1.17) (7). Biological mechanisms, mainly hyperinsulinemia and hyperglycemia, are linked to diabetes and can affect carcinogenesis (5, 8–10). Furthermore, some biological and behavioral factors such as age, sex, body weight, alcohol consumption, and smoking that were shown to associate with both diabetes and cancer, may also contribute to the relationship between these two diseases (4, 5, 8). Accordingly, all these findings infer an embedded association between cancer and diabetes (4–8).

Health is not limited to visiting a physician but also necessitates access to specific social and economic means for securing resources for quality medical care (11). Several types of determinants play a role in the risk of disease, including biological, genetic, behavioral, and social determinants (12). Research data determined that some indices of social determinants of health (SDOH) can affect both diabetes and cancer (13–15). For instance, depression, anxiety, and stress that emerge from living in low socioeconomic conditions, such as unfavorable housing, were shown to play a role in the increased risk of diabetes (16). Along the same lines, higher levels of education can result in higher-paying jobs and healthcare coverage, which in turn lead to better access to health information and awareness of cancer prevention strategies (15). Behavioral determinants of cancer such as alcohol consumption, physical inactivity, diet, smoking, occupational exposures, and cancer screening were also shown to be influenced by socioeconomic factors (17, 18).

Race was identified as an important associated factor for diabetes and cancer (4, 16, 19). For example, Asian men exhibited a higher risk of cancer incidence than non-Asian men (4). Additionally, people of the White race were less likely to develop diabetes than other racial groups (16), where, in the US, only 7.6% of White individuals have been diagnosed with diabetes compared to 15.9% of American Indians and Alaskan Natives, 13.2% of African Americans, and 12.8% of Hispanics (19).

Most of the available research conducted on diabetes and cancer assessed the epidemiologic, mechanistic, and biochemical links between these two diseases without considering the role of SDOH in this interplay. Measuring the impact of social factors remains important as socioeconomic standings in our society have resulted in health disparities and the information that can be generated from SDOH data may be used to refine cancer prevention and control tools (20). Therefore, the BRFSS (Behavioral Risk Factor Surveillance System) data is well-matched to address this gap in knowledge and offers a unique opportunity to determine the association of indices of SDOH and diabetes on the risk of developing cancer.

## 2. Methods

### 2.1. Study population and sampling

In this research work, we conducted a quantitative study using the 2020 BRFSS, a surveillance data collected yearly by the Centers for Disease Control and Prevention (CDC) on US residents in all the states (21), with questions relating to health risk behaviors, chronic health conditions, and use of preventive services. The reliability and validity of this surveillance data have been assessed and confirmed in several studies (22–24). A multistage cluster design was employed and adult participants (aged ≥18) were randomly selected by the CDC to provide a nationally representative sample (25). We excluded participants that reported having pre-diabetes or borderline diabetes, gestational diabetes, or missing data concerning their diabetes status. A total of 389,158 participants were included in this study.

### 2.2. Concepts and measures

The main dependent variable is cancer dichotomized into two categories indicating whether or not the participant has ever been diagnosed with cancer. The BRFSS data included a question on whether or not participants have been ever told that they have had any type of cancer. This question was used in our study as the outcome of interest to indicate presence or absence or cancer without specification of the type of cancer. Diabetes was also stratified into two levels indicating whether or not a participant has ever been diagnosed with diabetes. The indices of the SDOH considered in our study included homeownership, marital status, healthcare coverage, employment status, urban/rural county, education level, income level, and race. Our analysis was adjusted for additional biological and behavioral determinants of health that were known to be shared factors that associate with both diabetes and cancer (5). These covariates included age, sex, body-mass index (BMI), smoking status, and heavy alcohol consumption. The different categories of all the independent variables are displayed in Table 1.

**Table 1:** Characteristics of the study population.

Variables	*N*	Weighted %
*Cancer*		
Yes	62,579	11.1
No	324,859	88.5
*Missing*	*1,720*	*0.4*
*Diabetes*		
Yes	52,094	11.5
No	337,064	88.5
*Missing*	*–*	
*Cancer and diabetes*		
Yes	12,058	2.2
No	375,380	97.5
*Missing*	*1,720*	*0.3*
Social determinants of health		
*Home ownership*		
Own	272,109	66.1
Rent	93,936	26.5
Other arrangement	19,849	6.3
*Missing*	*3,264*	*1.1*
*Marital status*		
Married or coupled	215,785	54.7
Divorced or separated	57,583	12.8
Widowed	42,090	6.8
Never married	70,130	24.7
*Missing*	*3,570*	*1.0*
*Health care coverage*		
No	354,233	12.2
Yes	32,967	87.1
*Missing*	*1,958*	*0.7*
*Employment status*		
Employed or self-employed	195,294	54.7
Out of Work/Unable to Work	47,015	13.9
Homemaker/Student	26,788	10.1
Retired	113,289	19.2
*Missing*	*6,772*	*2.1*
*Urban/Rural county*		
Urban	325,044	92.6
Rural	57,371	6.3
*Missing*	*6,743*	*1.1*
*Education level*		
Did not graduate high school	25,114	12.3
Graduated high school	103,672	27.7
Attended college or technical school	107,691	30.4
Graduated college or technical school	150,942	29.1
*Missing*	*1,739*	*0.5*

Variables	*N*	Weighted %
*Income level*		
Less than 15,000$	25,468	7.4
15,000$ to less than 25,000$	46,894	12.0
25,000$ to less than 35,000$	30,285	7.4
35,000$ to less than 50,000$	42,501	10.1
More than 50,000$	166,857	42.5
*Missing*	*77,153*	*20.6*
*Race*		
White only	307,239	70.0
Black or African American only	30,813	12.5
Asian only	9,878	5.6
Other race only^1^	21,353	6.5
Multiracial	8,990	1.7
*Missing*	*10,885*	*3.7*
Additional variables		
*Age*		
18 to 44	117,897	45.6
45 to 54	55,676	14.7
55 to 64	74,779	16.2
65 or older	132,923	21.6
*Missing*	*7,883*	*1.9*
*Sex*		
Male	179,708	49.3
Female	209,450	50.7
*Missing*	*–*	
*Body-mass index (BMI)**^1^*		
Underweight	5,833	1.7
Normal Weight	107,513	28.0
Overweight	125,374	31.0
Obese	110,641	28.0
*Missing*	*39,797*	*11.3*
*Smoking status*		
Never smoked	217,503	58.0
Current smoker	50,688	13.3
Former smoker	100,558	22.4
*Missing*	*20,409*	*6.3*
Alcohol consumption^2^		84.7
Not a heavy drinker	336,121	
Heavy drinker	23,345	6.0
*Missing*	*29,692*	*9.3*

1 Other race included American Indian, Alaskan Native, Native Hawaiian/other Pacific Islander, or those who did not identify with any of the aforementioned race categories.

2 According to the CDC classification, BMI was classified into four categories: underweight (less than 18.5 kg/m^2^), normal weight (between 18.5 kg/m^2^ and 25.0 kg/m^2^), overweight (between 25.0 kg/m^2^ and 30.0 kg/m^2^), or obese (more than 30.0 kg/m^2^) (26, 27).

3 Heavy drinkers are defined as a man who consumes more than 14 drinks per week or a woman who consumes more than 7 drinks per week (21).

### 2.3. Statistical analysis

To adjust for the complex sampling design of the BRFSS, weighted analyses were conducted using sampling and cluster weights. Frequency and descriptive analyses were performed on all the variables included in this study. Counts and weighted percentages were generated. Unadjusted analysis was carried out to assess the crude association between SDOH and diabetes using weighted chi-square tests. Percentages and value of ps were reported. Adjusted analysis was also conducted using the weighted multiple logistic regression with diabetes as an outcome and the SDOH as predictors. Weighted adjusted odds ratios (ORs), their corresponding confidence intervals (CIs) and value of ps were all reported to determine the magnitude of association between all these factors and diabetes. Unadjusted analysis was performed using weighted simple logistic regression to assess the crude association between diabetes and SDOH as predictors, and cancer as the outcome of interest. Unadjusted ORs and 95% CIs were generated. Covariates that have value of ps ≤0.2 in the weighted simple logistic regression were considered eligible to be included in the adjusted analysis. In order to determine whether the indices of SDOH included in the multiple logistic regression were correlated, Cramer’s V was computed and reported for the different combinations of these determinants. Adjusted analysis was conducted using weighted multiple logistic regression where adjusted ORs and 95% CIs were generated. These analyses will determine whether the SDOH affect both diseases, and can therefore reinforce the triangulation between the social indices of health, diabetes and cancer.

## 3. Results

### 3.1. Descriptive statistics

Table 1 outlines the characteristics of the 389,158 participants included in this study from the 2020 BRFSS data. A low proportion of the participants (less than 20%) did not provide answers to some of the questions which were reflected in the missing values that we reported in Table 1. Our frequency distribution showed that the percent missing was very low (less than 10%) in majority of the variables (86%) that had missing values. With regard to the frequency distribution of our sample across all the variables, our results showed that 11.1% of the participants (*n* = 62,579) had cancer, 11.5% (*n* = 52,094) had diabetes, and 2.2% (*n* = 12,058) had both cancer and diabetes. Almost half of the participants were between the ages of 18 and 44 (45.6%, *n* = 117,897). The remaining were between the ages of 45 and 54 (14.7%, *n* = 55,676), 55 and 64 (16.2%, *n* = 74,779), and 65 or older (21.6%, *n* = 132,923). Nearly half of the participants were male (49.3%, *n* = 179,078), and the other half were female (50.7%, n = 209,450). Of the participants, 28.0% (*n* = 107,513) reported a BMI of normal weight, 1.7% (*n* = 5,833) were underweight, 31.0% (*n* = 125,374) overweight, and 28.0% (*n* = 110,641) were obese. Most participants never smoked (58.0%, *n* = 217,503) or stopped smoking (22.4%, *n* = 100,558), and did not consume alcohol heavily (84.7%, *n* = 336,121). Majority of the participants owned their home (66.1%, *n* = 272,109), were married (54.7%, *n* = 215,785), had healthcare coverage (87.1%, *n* = 354,233), and lived in urban counties (92.6%, *n* = 325,044). 54.7% (*n* = 195,294) of the participants were employed or self-employed, 13.9% (*n* = 47,015) were out of work or unable to work, 10.1% (*n* = 26,788) homemakers or students, and 19.2% (*n* = 113,289) retired. As for educational level, 27.7% of the participants graduated high school (*n* = 103,672), 12.3% (*n* = 25,114) did not finish high school, 30.4% (*n* = 107,691) attended college or technical school, and 29.1% (*n* = 166.857) graduated college or technical school. More than half of the participants had an income of more than $50,000 (42.5%, *n* = 166,857), 10.1% (*n* = 42,051) between $35,000 and $50,000, 7.4% (*n* = 30,285) between $25,000 and $35,000, 12.0% (*n* = 46,894) between $15,000 and $25,000, and 7.4% (*n* = 25,468) less than $15,000. Majority of the participants reported their race as White (70.0%, *n* = 307,239), 12.5% (*n* = 30,813) as Black or African American, 5.6% (*n* = 9,878) as Asian, 1.7% (*n* = 8,990) as multiracial, and 6.5% (*n* = 21,353) reported being of another race.

### 3.2. Distribution of cancer, SDOH, and other covariates among diabetics

Table 2 and Supplementary Table S1, respectively, display the frequency distribution of cancer, indices of the SDOH, and the other covariates among participants with diabetes and the respective unadjusted and adjusted associations between diabetes, SDOH, and the additional covariates. Our data showed that around 20% of diabetics had cancer (n = 12,058, *p* < 0.001), and that the SDOH and other covariates had significant unadjusted associations with diabetes (*p* < 0.001; Table 2). In addition, our adjusted analysis also showed that most of the SDOH were significantly associated with diabetes (Supplementary Table S1). The frequency distribution of the social factors among diabetics showed that most diabetics owned their home (73.26%, *n* = 37,685), were married (55.92%, *n* = 26,913), had healthcare coverage (92.56%, *n* = 49,237), and lived in urban areas (92.17%, *n* = 42,262). Concerning employment, 33.41% (*n* = 15,258) of diabetics were employed or self-employed, 39.21% (*n* = 24,148) were retired, 21.83% (*n* = 9,832) were out of work, and only 5.55% (*n* = 2,185) were homemakers or students. With respect to the level of education, most of the participants with diabetes attended college/technical school (30.99%, *n* = 15,434), or did not graduate high school (29.2%, *n* = 5,502), and 37.7% (*n* = 15,949) reported a high level of income of more than $50,000. Furthermore, the White race was highly prevalent among diabetics (69.09%, n = 38,908), followed by African Americans (17.85%, *n* = 6,098). Around half of the diabetic participants were aged 65 or more (45.25%, *n* = 28,561), were obese (54.07%, *n* = 25,198), and never smoked (52.78%, *n* = 25,627). Finally, the majority of diabetics were classified as being non-heavy drinkers (97.05%, *n* = 47,478).

**Table 2:** Frequency distribution of cancer, SDOH, and other covariates among diabetics, along with the respective associations between all these variables and diabetes.

Variables	*N*	Weighted %	*p*-value^ϯ^^*^
Cancer			
Yes	12,058	19.49	
No	39,698	80.51	<0.001
Social determinants of health			
*Home ownership*			
Own	37,685	73.26	
Rent	11,903	22.92	<0.001
Other arrangement	2,151	3.82	
*Marital status*			
Married or coupled	26,913	55.92	
Divorced or separated	9,678	17.7	<0.001
Widowed	9,280	14.14	
Never married	5,913	12.24	
*Health care coverage*			
Yes	49,237	92.56	
No	2,672	7.44	<0.001
*Employment status*			
Employed or self-employed	15,258	33.41	
Out of work/Unable to work	9,832	21.83	<0.001
Homemaker/Student	2,185	5.55	
Retired	24,148	39.21	
Urban/Rural county			
Urban	42,262	92.17	
Rural	8,688	7.83	<0.001
*Education level*			
Did not graduate high school	5,502	20.5	
Graduated high school	15,980	29.2	<0.001
Attended college or technical school	15,434	30.99	
Graduated college or technical school	14,978	19.3	
*Income level*			
Less than 15,000$	5,600	15.79	
15,000$ to less than 25,000$	8,987	20.86	<0.001
25,000$ to less than 35,000$	5,031	11.34	
35,000$ to less than 50,000$	6,152	14.24	
More than 50,000$	15,949	37.77	
*Race*			
White only	38,908	69.09	
Black or African American only	6,098	17.85	<0.001
Asian only	982	3.64	
Other race only	3,464	7.67	
Multiracial	1,265	1.74	

Variables	*N*	Weighted %	*p*-value^ϯ^^*^
Additional variables			
*Age*			
18 to 44	3,884	12.92	
45 to 54	6,327	15.62	<0.001
55 to 64	12,499	26.21	
65 or older	28,561	45.25	
*Sex*			
Male	24,991	50.77	
Female	27,103	49.23	<0.001
*Body-mass index (BMI)*			
Underweight	325	0.88	
Normal Weight	6,698	14.77	
Overweight	14,845	30.28	<0.001
Obese	25,198	54.07	
*Smoking status*			
Never smoked	25,627	52.78	
Current smoker	6,631	14.4	
Former smoker	17,384	32.82	<0.001
*Alcohol consumption*			
Not a heavy drinker	47,478	97.05	
Heavy drinker	1,366	2.95	<0.001

ϯ Weighted Chi-square test was conducted.

*All value of *ps* were ≤ 0.05 indicating significant associations.

### 3.3. Diabetes, SDOH, other covariates, and cancer

Supplementary Table S2 and Table 3 present the respective unadjusted and adjusted associations between diabetes, SDOH, and the additional covariates as predictors, and cancer as the outcome that were generated from the weighted simple and multiple logistic regression analyses. The study population included participants with and without diabetes so duration of diabetes was not accounted for in our main analysis since it necessitates that the study population encompass participants with diabetes only. Nonetheless, in a subsequent sub-analysis on diabetics only whereby the duration of diabetes was accounted for, our results showed a non-significant association between duration of diabetes and cancer with value of *p* 0.0302 (results not reported).

**Table 3:** Adjusted associations between diabetes, SDOH, other covariates, and cancer.

Cancer (outcome)	Weighted adjusted OR (95% CI)	*p*-value
*Diabetes*		
No	Ref	
Yes	1.13 (1.05–1.21)*	<0.001
Social determinants of health		
*Home ownership*		
Own	Ref	
Rent	0.86 (0.79–0.93)*	<0.001
Other arrangement	0.88 (0.75–1.04)	0.14
*Marital status*		
Married or coupled	Ref	
Divorced or separated	1.03 (0.95–1.11)	0.52
Widowed	1.01 (0.93–1.10)	0.81
Never married	0.73 (0.65–0.81)*	<0.001
*Health care coverage*		
No	Ref	
Yes	1.44 (1.22–1.71)*	<0.001
*Employment status*		
Employed or self-employed	Ref	
Out of work/Unable to work	1.78 (1.59–1.99)*	<0.001
Homemaker/student	1.05 (0.90–1.23)	0.53
*Retired*	1.54 (1.43–1.67)*	<0.001
*Urban/rural county*		
Urban	Ref	
Rural	1.06 (0.99–1.13)	0.10
*Education level*		
Graduated high school	Ref	
Did not graduate high school	1.05 (0.89–1.22)	0.57
Attended college or technical school	1.10 (1.02–1.18)*	0.01
Graduated college or technical school	1.31 (1.21–1.40)*	<0.001
*Income level*		
Less than 15,000$	Ref	
15,000$ to less than 25,000$	1.16 (1.02–1.32)*	0.02
25,000$ to less than 35,000$	1.17 (1.01–1.36)*	0.03
35,000$ to less than 50,000$	1.32 (1.12–1.54)*	<0.001
More than 50,000$	1.38 (1.21–1.58)*	<0.001
*Race*		
White only	Ref	
Black or African American only	0.44 (0.39–0.49)*	<0.001
Asian only	0.27 (0.20–0.38)*	<0.001
Other race only	0.56 (0.46–0.69)*	<0.001
Multiracial	0.80 (0.61–1.04)	0.09

Cancer (outcome)	Weighted adjusted OR (95% CI)	*p*-value
Additional variables		
*Age*		
18 to 44	Ref	
45 to 54	2.91 (2.55–3.31)*	<0.001
55 to 64	5.50 (4.89–6.19)*	<0.001
65 or older	10.70 (9.47–12.09)*	<0.001
*Sex*		
Male	Ref	
Female	1.14 (1.08–1.21)*	<0.001
*Body-Mass Index (BMI)*		
Normal weight	Ref	
Underweight	1.25 (1.00–1.57)*	0.05
Overweight	0.94 (0.88–1.01)	0.08
Obese	0.99 (0.92–1.06)	0.73
*Smoking status*		
Never smoked	Ref	
Current smoker	1.71 (1.07–1.28)*	<0.001
Former smoker	1.26 (1.18–1.33)*	<0.001
*Alcohol consumption*		
Not a heavy drinker	Ref	
Heavy drinker	1.09 (0.97–1.22)	0.13

*p-value ≤ 0.05 indicating significant results.

The indices of the SDOH that were eligible to be included in the weighted multiple logistic regression (Table 3) were home ownership, marital status, healthcare coverage, employment status, urban/rural county, education level, income level, and race. The aforementioned SDOH had low correlations with Cramer’s V being less than 0.3 between all the SDOH indices (Supplementary Table S3). Diabetics exhibited a 13% increase in the odds of cancer (OR 1.13; 95%CI: 1.05–1.21) compared to non-diabetics (Table 3). This increase in the odds of cancer indicates that individuals with diabetes could be at a higher risk of developing cancer which then necessitates that they undergo more frequent screening and preventative measures for cancer compared to non-diabetics. People who rented their homes had 14% lower odds of cancer (OR 0.86; 95%CI: 0.79–0.93) than homeowners, and participants who were never married had 27% lower odds of cancer (OR 0.73; 95%CI: 0.65–0.81) compared to those who were married. Participants with healthcare coverage showed 44% greater odds of cancer (OR 1.44; 95%CI: 1.22–1.71) than those without. Unemployed (OR 1.78; 95% CI: 1.59–1.99) and retired individuals (OR 1.54; 95% CI: 1.43–1.67) had almost double the odds of cancer relative to those who are employed. Participants who attended or graduated college had 10–31% higher odds of cancer (OR 1.10; 95%CI: 1.02–1.18 & OR 1.31; 95%CI: 1.21–1.40, respectively) compared to individuals who only graduated high school. Our results also indicated that participants whose income was in higher ranges had a significant increase in the odds of cancer that ranged between 1.16 and 1.38 when compared to those whose annual income was less than $15,000. Black or African-Americans had 56% lower odds of cancer compared to White individuals (OR 0.44; 95%CI: 0.39–0.49). The same was denoted for Asians (OR 0.27; 95%CI: 0.20–0.38), and other races (OR 0.56; 95%CI: 0.46–0.69) which had, respectively, 73 and 44% decreased odds of cancer compared to White individuals. These reported odds do not necessarily indicate increased or decreased risk of cancer, rather they could be reflective of the frequency of diagnosis and access to screening facilities among individuals with specific indices of SDOH.

Older age categories exhibited greater odds of cancer which may imply that people of older age are at a greater risk of developing cancer. In specific, participants whose ages ranged from 45 to 54 had almost triple the odds of cancer (OR 2.91; 95%CI: 2.55–3.31), while those who were between 55 to 64 years of age had approximately 6 times the odds of cancer (OR 5.50; 95%CI: 4.89–6.19), and those who were 65 and older had 11 times the odds of cancer (OR 10.70; 95%CI: 9.47–12.09), compared to the younger age group of 18 to 44 years. Females showed 14% higher odds of cancer (OR 1.14; 95%CI: 1.08–1.21) compared to males, which could be due to inherent genetic and/or biologic factors that increase the risk of cancer among females. Similarly, underweight participants had a 25% increase in the odds of cancer (OR 1.25; 95%CI: 1.00–1.57) compared to participants with normal weight. This observation may be due to the fact that people with cancer tend to experience unintentional weight loss that is either triggered by the cancer itself or resulting from the fatigue and loss of appetite that accompany tumor treatment regimens. Lastly, smoking was shown to be a risk factor for cancer with current smokers (OR 1.71; 95%CI: 1.07–1.28) and former smokers (OR 1.26; 95%CI: 1.18–1.33) having increased odds of cancer of 71 and 26%, respectively, compared to those who never smoked, a result that has been long established identifying smoking as a risk factor for cancer.

## 4. Discussion

Our findings highlighted the association between diabetes and cancer, whereby diabetics showed an excess risk of cancer by 13%. This is consistent with other reported results which determined 10–16% increase in the risk of cancer among people with diabetes (3, 4, 6, 7, 9, 10). The possible biological links between the two diseases are hyperinsulinemia and hyperglycemia, with hyperglycemia being a causative factor on its own for cancer, or a surrogate for hyperinsulinemia established to be responsible for increased tumor growth (5, 28). Our results showed that age, sex, BMI, and smoking were associated with cancer and diabetes corroborating their role as shared associated factors between diabetes and cancer and explaining the relationship between the two diseases (4, 5, 8).

Furthermore, we also assessed the interplay between indices of SDOH, diabetes, and cancer, and revealed the triangulation between the social indices of health and these diseases. In this respect, our results showed that the SDOH were significantly linked with and affected both diabetes and cancer. While previous studies have reported an effect of SDOH on diabetes and cancer independently, we presented in this study a comprehensive analysis of the relationship between SDOH, diabetes, and cancer combined and highlighted the socioeconomic and racial inequities in the distribution of diabetes and cancer in the US. Social factors that were associated with increased odds of cancer included employment status as being out of work/unable to work, and retired, education level as attended college/technical school, graduated college/technical school, higher income levels, healthcare coverage, and white race (compared to all other races); while the SDOH that showed a decrease in the odds of cancer included renting a home and never been married. These results highlight the interplay between diabetes, SDOH, and cancer, and confirm the role of diabetes and some indices of SDOH as risk factors for cancer.

Our results regarding the employment status which indicated that being out of work/unable to work or retired had higher odds of cancer compared to employed/self-employed were anticipated. If we consider for instance the retired group, we can link their increased odds of cancer to probably their older age and/or their physical inability to work (5, 29). Along the same lines, one can also relate the inability to work or being out of work to physical barriers that preclude individuals from resuming their daily job activities. This observation is in line with the literature which reported that people diagnosed with cancer and are undergoing treatment are less likely to get employed or maintain their jobs (30).

Based on all these findings, one can deduce that improved social conditions reflected in owning a home, having higher income, healthcare coverage, and higher education were all social indices that demonstrated significant links with cancer. Marriage can be also considered an indicator of higher socioeconomic status as it is associated with financial stability (31, 32). White people as well, who, in the US, live in significantly improved social conditions compared to other races, are considered among those in the high socioeconomic levels (17).

These socioeconomic and racial disparities can be interpreted in relation to the healthcare system and in relation to behavioral and psychological factors. In this regard, multiple studies showed that social determinants of health contribute to up to 70% of cancer cases, increased risk of death, and reduced survival rates. This association could be explained through different indices of SDOH that include education, housing arrangements, income among others. In this regard educated people tend to lead healthier lifestyle and are less likely to be exposed to hazardous occupational substances, whereas having living arrangements that are unstable are linked to reduced access to healthcare services and consequently, late diagnosis of tumor and poor health outcome. Behavioral and biologic factors such as smoking, alcohol consumption, obesity, and lack of access to healthy food all of which affect disproportionally lower socio-economic groups were also shown to associate with higher rates of cancer (33).

Previous research has shown that differences in the use of screening programs and early diagnosis are the most plausible explanations for the higher incidence of cancer in the least deprived socioeconomic population groups (34–36). This is reflected by the fact that people who live in the most deprived neighborhoods show a substantially increased rate of late-stage cancer diagnosis (37). Another example is Black women, where this group of females tends to have lower access to routine screening reflecting hesitancies in their attitude toward preventative measures compared to White women and consequently advanced stages of detected cancer (20, 38). Even in countries where universal healthcare coverage is present, social disparities in the use of screening procedures exist (34, 39, 40). Therefore, high socioeconomic status is related to better engagement in medical testing and follow-up. Education and accessibility to diabetes and cancer prevention and control instruments lead to improved efforts for monitoring possible complications of diabetes and screening for cancer because where these conditions are present, healthcare systems have an improved capacity to identify and follow up with people at risk for disease.

Behavioral and psychological factors may also be the intermediating steps between SDOH and health outcomes (14). Higher socioeconomic groups have been shown to have a more sedentary life, lower physical activity, and more consumption of red meat and high-fat food, all of which are behavioral determinants of cancer (14, 41). While most of these behaviors can be considered individually, they are strongly tied to the social context (14). For example, social approaches to control tobacco smoking are more effective than individual-level approaches (42–44). Furthermore, people of higher social status have better access to health information which leads to better engagement in healthcare and timely detection of cancer (15). Early detection procedures, a secondary prevention strategy for cancer, mainly rely on the behaviors of individuals and their attitudes toward their health and well-being (14, 26, 43). However, socioeconomic circumstances also play a significant role in the choice of and adherence to cancer treatment, as well as the quality of life and burden of symptoms (39, 44). Our findings showed a negative association between cancer diagnosis and factors such as renting a home, never being married, and belonging to certain racial groups such as African Americans, Asians, and other races compared to whites. These observations might be reflective of non-adherence to preventative medical examination and routine check-ups that may be related to financial (34–37) and/or behavioral (15) barriers among this subpopulation. Nevertheless, lower detection of cancer does not necessarily translate into better disease outcomes. On the contrary, a higher cancer survival rate was detected among individuals with earlier diagnosis and higher reporting of cancer which mostly occur in higher socioeconomic status (34, 37) and white race (37). Accordingly, this calls for better policymaking to ensure more equity in healthcare coverage and health-related services.

## 5. Limitation

There are factors that we opted to include in our analysis and which may have had a confounding effect on cancer but the BRFSS did not collect information on. These covariates include genetic testing, family history, exposure to hazardous material, trust in the healthcare system, perceived quality of care, and satisfaction with how information are being communicated by the healthcare providers among other determinants. These factors need to be explored in future studies to provide a more in-depth understanding of all the determinants that may be linked to cancer. The generalizability of the results to the entire population of the US may be limited due to the BRFSS including participants who have access to cellular or landline telephones, thereby excluding individuals without such access. Second, the data collected through the BRFSS are self-reported, which introduces potential issues related to recall bias. However, despite these limitations, the BRFSS data remains reliable and valid. The BRFSS adopts weighting methods to minimize response bias and improve the alignment of the sample distribution with the demographic characteristics of state populations (22, 45).

## 6. Conclusion

Our study revealed that diabetics are a high-risk population for cancer. Our findings also showed that some indices of SDOH, such as home ownership, marital status, healthcare coverage, employment status, education level, income level, and race can be associated with cancer. To our knowledge, this is the first study to triangulate the relationship between the two diseases and SDOH. Defining the basis of social disparities in cancer and understanding the role of each social determinant could lead to specific actions to address social inequities in disease prevention and access to healthcare. The available evidence can support some potential strategies such as free testing, improved health communication, and increased engagement with primary-care physicians for lower socioeconomic groups. Social inequities are avoidable and therefore their reduction should be considered an achievable goal. Our results can inform targeted public health interventions and strategies to reduce the burden of cancer, especially among individuals with diabetes. In this respect, healthcare providers and public health officers and agencies should adopt a holistic approach, recognizing the role of social determinants such as income, education, and healthcare access when developing public health strategies. Additionally, future research should focus on understanding the mechanisms linking diabetes and cancer, exploring the effectiveness of interventions targeting social determinants and evaluating the interventions addressing socioeconomic factors to improve health outcomes and reduce social and racial disparities in health.

## Data availability statement

The original contributions presented in the study are included in the article/Supplementary material, further inquiries can be directed to the corresponding authors.

## Ethics statement

Ethical review and approval was not required for the study on human participants in accordance with the local legislation and institutional requirements. The patients/participants provided their written informed consent to participate in this study.

## Author contributions

NM and MJ conceived the research questions. AJ and HT refined and finalized the conceptual ideas. NM carried out the data analysis and drafted the manuscript. MJ oversaw the data analysis. AJ, HT, and MJ revised and edited the manuscript. NM, AJ, HT, and MJ all approved the final version of the manuscript. All authors contributed to the article and approved the submitted version.

## Conflict of interest

The authors declare that the research was conducted in the absence of any commercial or financial relationships that could be construed as a potential conflict of interest.

## Publisher’s note

All claims expressed in this article are solely those of the authors and do not necessarily represent those of their affiliated organizations, or those of the publisher, the editors and the reviewers. Any product that may be evaluated in this article, or claim that may be made by its manufacturer, is not guaranteed or endorsed by the publisher.

## Abbreviations

BMI: Body-Mass Index
BRFSS: Behavioral Risk Factor Surveillance System
CDC: Centers for Disease Control and Prevention
CI: Confidence Interval
NCDs: Non-Communicable Diseases
OR: Odds Ratio
RR: Relative Risk
SDOH: Social Determinants of Health
US: United States
