# Supplementary material for: Social and racial inequalities in diabetes and cancer in the United States
Source: Front Public Health. 2023 Jul 19;11:1178979. doi: 10.3389/fpubh.2023.1178979 (PMC10395076; doi:10.3389/fpubh.2023.1178979)
Supplement: Supplementary file 1 [file Table_1.DOCX]

**Supplementary Table S1 Adjusted Associations between SDOH, Other Covariates, and Diabetes**

| **Diabetes (outcome)** | **Weighted Adjusted OR (95% CI)** | **p-value** |
| --- | --- | --- |
| **Social Determinants of Health** |  |  |
| Home Ownership  *Own*  *Rent*  *Other Arrangement* | Ref  1.04 (0.95-1.13)  0.90 (0.75-1.07) | 0.38  0.24 |
| Marital Status  *Married or Coupled*  *Divorced or Separated*  *Widowed*  *Never Married* | Ref  0.88 (0.81-0.96)*  1.02 (0.93-1.13)  0.77 (0.69-0.86)* | 0.004*  0.61  <0.001* |
| Health Care Coverage  *No*  *Yes* | Ref  1.54 (1.33-1.78)* | <0.001* |
| Employment Status  *Employed or Self-Employed*  *Out of Work/Unable to Work*  *Homemaker/Student*  *Retired* | Ref  1.78 (1.62-1.97)*  1.07 (0.92-1.25)  1.47 (1.34-1.60)* | <0.001*  0.35  <0.001* |
| Urban/Rural County  *Urban*  *Rural* | Ref  1.02 (0.94-1.09) | 0.67 |
| Education Level  *Graduated high school*  *Did not graduate high school*  *Attended college or technical school*  *Graduated college or technical school* | Ref  1.32 (1.17-1.48)*  1.05 (0.97-1.13)  0.79 (0.74-0.86)* | <0.001*  0.22  <0.001* |
| Income Level  *Less than 15,000$*  *15,000$ to less than 25,000$*  *25,000$ to less than 35,000$*  *35,000$ to less than 50,000$*  *More than 50,000$* | Ref  0.82 (0.73-0.93)*  0.80 (0.69-0.92)*  0.73 (0.63-0.84)*  0.54 (0.47-0.62)* | 0.002*  0.002*  <0.001*  <0.001* |
| Race  *White only*  *Black or African American only*  *Asian only*  *Other race only*  *Multiracial* | Ref  1.64 (1.49-1.80)*  1.62 (1.33-1.98)*  1.50 (1.27-1.78)*  1.35 (1.05-1.74)* | <0.001*  <0.001*  <0.001*  0.02* |

| ***Supplementary Table S1 Adjusted Associations between SDOH, Other Covariates, and Diabetes (Continued)*** | | | |
| --- | --- | --- | --- |
|  | |  |  |
| **Diabetes (outcome)** | **Weighted Adjusted OR (95% CI)** | | **p-value** |
| **Additional Variables** | |  |  |
| Age  *18 to 44*  *45 to 54*  *55 to 64*  *65 or older* | | Ref  3.33 (2.96-3.75)*  5.23 (4.67-5.86)*  6.98 (6.18-7.89)* | <0.001*  <0.001*  <0.001* |
| Sex  *Male*  *Female* | | Ref  0.78 (0.73-0.83)* | <0.001* |
| Body-Mass Index (BMI)  *Normal Weight*  *Underweight*  *Overweight*  *Obese* | | Ref  0.90 (0.63-1.28)  1.69 (1.54-1.85)*  4.14 (3.79-4.53)* | 0.55  <0.001*  <0.001* |
| Smoking Status  *Never Smoked*  *Current Smoker*  *Former Smoker* | | Ref  1.08 (0.99-1.18)  1.11 (1.04-1.19)* | 0.08  0.002* |
| Alcohol Consumption  *Not a Heavy Drinker*  *Heavy Drinker* | | Ref  0.51 (0.44-0.61)* | <0.001* |

*p-value ≤ 0.05 indicating significant results
