# Supplementary material for: Social and racial inequalities in diabetes and cancer in the United States
Source: Front Public Health. 2023 Jul 19;11:1178979. doi: 10.3389/fpubh.2023.1178979 (PMC10395076; doi:10.3389/fpubh.2023.1178979)
Supplement: Supplementary file 2 [file Table_2.DOCX]

**Supplementary Table S2 Unadjusted Associations between Diabetes, SDOH, Other Covariates, and Cancer**

| **Cancer (outcome)** | **Weighted Unadjusted OR (95% CI)** | **p-value** |
| --- | --- | --- |
| Diabetes  *No*  *Yes* | Ref  2.16 (2.04-2.28)* | <0.001 |
| **Social Determinants of Health** |  |  |
| Home Ownership  *Own*  *Rent*  *Other Arrangement* | Ref  0.32 (0.30-0.33)*  0.28 (0.25-0.32)* | <0.001  <0.001 |
| Marital Status  *Married or Coupled*  *Divorced or Separated*  *Widowed*  *Never Married* | Ref  1.03 (0.97-1.09)  2.35 (2.21-2.50)*  0.23 (0.22-0.25)* | 0.29  <0.001  <0.001 |
| Health Care Coverage  *No*  *Yes* | Ref  3.82 (3.36-4.34)* | <0.001 |
| Employment Status  *Employed or Self-Employed*  *Out of Work/Unable to Work*  *Homemaker/Student*  *Retired* | Ref  1.78 (1.65-1.92)*  0.78 (0.70-0.88)*  6.03 (5.76-6.31)* | <0.001  <0.001  <0.001 |
| Urban/Rural County  *Urban*  *Rural* | Ref  1.31 (1.25-1.39)* | <0.001 |
| Education Level  *Graduated high school*  *Did not graduate high school*  *Attended college or technical school*  *Graduated college or technical school* | Ref  0.95 (0.86-1.05)  1.15 (1.09-1.22)*  1.23 (1.17-1.29)* | 0.29  <0.001  <0.001 |
| Income Level  *Less than 15,000$*  *15,000$ to less than 25,000$*  *25,000$ to less than 35,000$*  *35,000$ to less than 50,000$*  *More than 50,000$* | Ref  1.16 (1.05-1.29)*  1.24 (1.10-1.39)*  1.37 (1.23-1.53)*  1.21 (1.10-1.32)* | <0.001  <0.001  <0.001  <0.001 |
| Race  *White only*  *Black or African American only*  *Asian only*  *Other race only*  *Multiracial* | Ref  0.34 (0.31-0.37)*  0.15 (0.11-0.20)*  0.34 (0.29-0.39)*  0.52 (0.43-0.63)* | <0.001  <0.001  <0.001  <0.001 |

| ***Supplementary Table S2 Unadjusted Associations between Diabetes, SDOH, Other Covariates, and Cancer (Continued)*** | | | |
| --- | --- | --- | --- |
|  | |  |  |
| **Cancer (outcome)** | **Weighted Unadjusted OR (95% CI)** | | **p-value** |
| **Additional Variables** | |  |  |
| Age  *18 to 44*  *45 to 54*  *55 to 64*  *65 or older* | | Ref  3.71 (3.35-4.12)*  7.96 (7.27-8.72)*  19.17 (17.65-20.83)* | <0.001  <0.001  <0.001 |
| Sex  *Male*  *Female* | | Ref  1.23 (1.18-1.28)* | <0.001 |
| Body-Mass Index (BMI)  *Normal Weight*  *Underweight*  *Overweight*  *Obese* | | Ref  1.07 (0.91-1.27)  1.13 (1.07-1.19)*  1.06 (1.01-1.12)* | 0.41  <0.001  0.03 |
| Smoking Status  *Never Smoked*  *Current Smoker*  *Former Smoker* | | Ref  1.02 (0.95-1.09)  2.09 (1.99-2.19)* | 0.57  <0.001 |
| Alcohol Consumption  *Not a Heavy Drinker*  *Heavy Drinker* | | Ref  0.99 (0.90-1.08) | 0.81 |

*p-value ≤ 0.05 indicating significant results
