# Supplementary material for: Social and racial inequalities in diabetes and cancer in the United States
Source: Front Public Health. 2023 Jul 19;11:1178979. doi: 10.3389/fpubh.2023.1178979 (PMC10395076; doi:10.3389/fpubh.2023.1178979)
Supplement: Supplementary file 3 [file Table_3.docx]

**Supplementary Table S3 Measures of Correlation between all the Indices of SDOH Assessed by Cramer's V**

| **SDOH** | Home Ownership | Marital Status | Healthcare Coverage | Employment Status | Urban/Rural Status | Education Level | Income Level | Race |
| --- | --- | --- | --- | --- | --- | --- | --- | --- |
| Home Ownership |  | 0.3043 | 0.1828 | 0.2065 | 0.0825 | 0.1419 | 0.2397 | 0.1505 |
| Marital Status |  |  | 0.1225 | 0.2273 | 0.0606 | 0.00961 | 0.2306 | 0.1074 |
| Healthcare Coverage |  |  |  | 0.1663 | -0.0015 | 0.1935 | 0.1773 | 0.1042 |
| Employment Status |  |  |  |  | 0.0433 | 0.1293 | 0.2489 | 0.0925 |
| Urban/Rural Status |  |  |  |  |  | 0.0863 | 0.0797 | 0.0886 |
| Education Level |  |  |  |  |  |  | 0.2545 | 0.0914 |
| Income Level |  |  |  |  |  |  |  | 0.0962 |
